# Modern Breast Cancer Detection: A Technological Review

**DOI:** 10.1155/2009/902326

**Published:** 2009-12-28

**Authors:** Adam B. Nover, Shami Jagtap, Waqas Anjum, Hakki Yegingil, Wan Y. Shih, Wei-Heng Shih, Ari D. Brooks

**Affiliations:** ^1^Department of Surgery, Drexel University College of Medicine, Philadelphia, PA 19102, USA; ^2^Department of Materials Science and Engineering, College of Engineering, Drexel University, Philadelphia, PA 19104, USA; ^3^School of Biomedical Engineering, Science & Health Systems, Drexel University, Philadelphia, PA 19104, USA

## Abstract

Breast cancer is a serious threat worldwide and is the number two killer of women in the United States. The key to successful management is screening and early detection. What follows is a description of the state of the art in screening and detection for breast cancer as well as a discussion of new and emerging technologies. This paper aims to serve as a starting point for those who are not acquainted with this growing field.

## 1. Introduction

Breast cancer is the most common form of cancer among women and the second most common cancer in the world (an estimated 1 152 161 new cases per year), trailing only lung cancer [1]. In 2009, an estimated 40 610 people (men and women) were projected to die of breast cancer in the United States [2]. The current approach to this disease involves early detection and treatment. This approach in the United States yields an 85% 10-year survival rate. Survival is directly related to stage at diagnosis, as can be seen by a 98% 10-year survival rate for patients with stages 0 and I disease compared with a 65% 10-year survival rate for patients with stage III disease. To improve survival in this disease, more patients need to be identified at an early stage. Therefore, we evaluated existing and emerging technologies used for breast cancer screening and detection to identify areas for potential improvement. The main criteria for a good screening test are accuracy, high sensitivity, acceptable specificity, ease of use, acceptability to the population being screened (with regard to discomfort and time), and low cost. This paper begins by describing commonly used breast cancer detection techniques and then delves into emerging modalities.

## 2. Commonly Used Methods

### 2.1. Breast Self-Examinations, Clinical Breast Examinations, and Mammography

Breast self-examination (BSE) and clinical breast examination (CBE) are used to screen for breast cancer. CBE has a sensitivity of 57.14% and a specificity of 97.11% [3]. Although it does not permit one to determine malignancy with assurance, it is useful for detecting suspicious breast lesions. Kosters and Gotzsche [4] found no improvement in breast cancer mortality rates in those screened using BSE and CBE compared with those with no screening, although those screened underwent twice as many biopsies. Despite these results and those from similar studies, some believe that CBE and BSE should still be used, especially for women younger than 40 years as well as for those who do not undergo routine mammography [5]. Other studies indicate that many teachers and nurses, that is, those who influence young women, are not fluent in BSE, either because they are uneducated on the subject or because they fail to perform it correctly [6]. In one study, 99% of nurse participants felt capable of performing a BSE, yet only 26% performed a monthly BSE [7]. A definite need exists to improve our ability to teach and validate BSE and CBE so that these methods can be used effectively for screening.

Generally referred to as the gold standard of breast imaging, mammography, or screen-film mammography (SFM), is the most common form of breast imaging. It is basically an X-ray examination of the breast under compression. It has a true-positive rate of 83% to 95% and a false-positive rate of 0.9% to 6.5% (note, however, that these figures stem from meta-analysis, which uses an idealized population of 27% young women, while the false-positive rate in young women is known to be higher) [8]. Sensitivity and specificity of mammography are affected by breast density, which in turn is affected by “age, use of hormone replacement therapy (HRT), menstrual cycle phase, parity, body mass index, and familial or genetic tendency” [8, 9]. In one study, sensitivity was 68.6% in women aged 40 to 44 and 83.3% in women aged 80 to 89; whereas specificity values (in women who did not use hormone replacement therapy) were 91.4% and 94.4%, respectively [9]. In that study, the results from women of all ages who used hormone replacement therapy yielded a mammographic specificity of about 91.7% [9]. Mammography is less sensitive in women with radiographically dense breasts. Sensitivity values range from 62.9% in extremely dense-breasted women to 87% in extremely fatty-breasted women, whereas specificity values ranged from 89.1% to 96.9%, respectively [9].

Mammograms have certain limitations. They require a dedicated machine, radiographic film and developing chemicals, a trained X-ray technologist, and a radiologist to read the films. They require breast compression, which causes the patient discomfort. Images seen on mammograms also lead to unnecessary biopsies. A study showed that biopsies of microcalcifications seen on mammograms yielded only 36.5% containing malignant tissue [10]. The process also exposes the breast to radiation, a “mean glandular dose from 2-view mammography of approximately 4 to 5 mGy” [11], which may cause radiation carcinogenesis. Despite this hazard, the benefits of a mammogram outweigh the risks [12]. As a screening modality, SFM remains the current standard for detecting breast cancer. The technology has been in place for more than 30 years, and the images are widely accepted as standard in clinical use. Still, newer technologies may prove more favorable than mammography, and much research focuses on obtaining better systems for breast cancer detection.

### 2.2. Full-Field Digital Mammography

Full-field digital mammography (FFDM) is simply a digital mammogram. Rather than recording an image on film, FFDM records an image in an electronic file. Mammography is divided into discrete processes: image acquisition, image display, and image storage, so each can be optimized separately [13]. FFDM allows real-time image presentation, postimage processing, and digital storage [14, 15]. Digital mammographic images can be transmitted electronically (e.g., via the Internet) in a process known as telemammography [13], allowing the radiologist and the clinician to review the images from a remote location. The machines are 10 to 40 times more expensive to buy than film screen mammography units but yield a cost savings by removing the need for film and developing chemicals as well as reducing the need for call backs to bring patients back due to poor image quality. FFDM does have disadvantages. It has poorer spatial resolution than film mammography, and its files require large amounts of digital storage space [15].

Cancer detection rates using FFDM or SFM are basically equal [16, 17], but FFDM allows better classification of lesions through the Breast Imaging Reporting and Data System (BI-RADS). One study found the sensitivity and specificity for FFDM to be 95.2% and 41.4%, respectively, in comparison to those for SFM, which were 91.9% and 39.3% [14]. A large-scale comparison study (over 40 000 participants), the Digital Mammographic Imaging Screening Trial (DMIST), by Pisano et al. [18] concluded that FFDM is more accurate in “women under the age of 50 years, women with radiographically dense breasts, and premenopausal or perimenopausal women.”

### 2.3. Computer-Aided Detection (CAD)

CAD is pattern recognition software that identifies suspicious abnormalities on images, marking them for the radiologist. CAD also stands for computer-aided diagnosis, which refers to a system that marks benign or malignant images, and the two acronyms are often confused. The most popular CAD system is the R2 Image Checker (R2 Technology, Inc., Santa Clara, CA), which combines detection and diagnosis [15].

The Food and Drug Administration requires radiologists to review and interpret FFDM data before seeing CAD marks [19]. Furthermore, because CAD does not mark all suspicious areas, radiologists must not disregard findings not also found by CAD [19]. Also, because CAD shows more false than true CAD marks, the radiologist must decide which marks are correctly placed [19]. In one study, 97.4% of CAD marks were rejected by the radiologist [20].

In a retrospective study done in 2000, radiologists had a false-negative rate of 21%, which could have been reduced by 77% with the use of CAD [21]. Freer and Ulissey [20] found that the introduction of CAD increased the recall rate from 6.5% to 7.7%, the number of cancers found by 19.5%, and the number of early-stage malignancies from 73% to 78%. This study also showed that CAD and radiologists had about the same accuracy: CAD detected 40 of 49 lesions, whereas the radiologist detected 41 of 49 lesions [20]. Brem et al. [22] found that CAD was more accurate in detecting microcalcifications than masses. She and her colleagues also found that CAD had high sensitivities for ductal carcinoma in situ (DCIS) and invasive lobular carcinoma. CAD systems have been in clinical use since 2000 and can be used directly on digital mammography files or on scanned SFM films. CAD can also be applied to other images such as those generated by computed tomography, ultrasound, and magnetic resonance imaging.

### 2.4. Modalities Using Ultrasound

Ultrasonography (US), also known as sonomammography, is frequently used to image palpable masses in the breast or as a follow-up test for abnormal results on a mammogram. The current technology involves a handheld transducer placed on the breast surface by a technologist and moved around to image the breast below the transducer. The images obtained must be labeled by the technologist in regard to location in the breast and orientation. A slight change in orientation of the beam may miss an area or image it twice, making US an operator-dependent modality. Lastly, the usual probe is 1 cm by 6 cm, which makes it difficult to cover the entire surface of a breast in a reliable fashion for screening.

#### 2.4.1. B-Mode Ultrasound

The most common form of ultrasound, B-mode, uses sound waves bounced off tissues to create an image of the breast. The strength of each echo determines the brightness of that point. From these echoes, two-dimensional images are created, generally about 30 images per second, allowing for real-time imaging [23]. Various tissues can be identified by brightness and image. Ultrasound is most often used to characterize lesions, to identify palpable masses that cannot be seen on mammograms, and to examine women not able to undergo mammography: the young and the pregnant [23]. It is also suitable for scanning dense breasts. B-mode ultrasound is also used to guide biopsies. Computer-aided diagnosis technology can be applied to US yet causes the loss of the real-time analysis that makes US so popular [23].

US is generally not used for screening, although some studies indicate otherwise. A study by Kolb et al. [24] shows a 17% increase in overall cancer detection and a 37% increase in tumors detected by imaging when US was added to a mammographic screening. A study by Rahbar [25] and colleagues indicates that US can aid in the differentiation between benign and malignant masses based on the shape of the mass's image. Simple cysts are diagnosed with 98% to 100% accuracy, yet more complex cysts yield a lower sensitivity [23]. Unfortunately, B-mode ultrasonographic images contain speckle and clutter, lowering the image quality [23, 26]. Also, lesion lateral margins are difficult to detect [23].

#### 2.4.2. Compound Imaging

Compound imaging combines multiple US images into one, decreasing the amount of speckle, clutter, noise, and shadowing, yielding a higher quality, “smoother,” image [23, 26, 27]. Compound imaging improves tissue differentiation, margin visualization, internal architecture visualization, and low-contrast lesion conspicuity, creating a more realistic image [26, 27]. Although better in quality than B-mode US, compound imaging is subject to blurring, and it suppresses shadows that can be used to determine malignancy [23].

#### 2.4.3. Doppler Ultrasonography

Doppler US uses the Doppler effect to track blood flow, finding malignant tumors through their neoangiogenesis. Two types of Doppler ultrasound exist: color and power, though power Doppler ultrasound is better for tracking intralesional blood flow [15, 28]. Cosgrove et al. [29] found that 99% of malignant lesions contained blood vessels and that 96% of benign lesions showed no color Doppler marks, indicating that color Doppler marks should warrant a biopsy. Despite this study and that of Raza and Baum [30], who found 68% sensitivity, 95% specificity, 85% positive predictive value, and 88% negative predictive value, other studies have reported less favorable views of Doppler US [23, 28]. Doppler US can be enhanced using microbubble-based contrast agents. Although regular Doppler US cannot depict blood vessels smaller than 100 to 200 *μ*m, smaller vessels can be seen with contrast enhancements, though various studies on the subject report conflicting results [23]. One study shows an increase in sensitivity from 36% to 95%, positive predictive values from 67% to 78%, and negative predictive values from 63% to 96%, although specificity decreased from 86% to 79% when Doppler was added to conventional sonography [31].

### 2.5. Magnetic Resonance Imaging (MRI)

MRI can create either 2- or 3D images, which, depending on the acquisition style, static or dynamic, have tradeoffs between spatial and temporal resolution [32, 33].

Magnetic resonance imaging uses a magnetic field (generally 1.0–1.5 T) and radio waves to change the alignment of hydrogen nuclei and, from this change, creates an image. A gadolinium-based contrast agent is commonly used in breast imaging [15, 33]. The subject lies prone, while either one or both breasts, depending on the type of coil, are imaged [33, 34]. Whereas mammography works best with fatty breasts, fat must be suppressed in MRI, either through postprocessing subtraction or other techniques before contrast agent injection, to preserve the image quality [33].

The sensitivity of MRI in visualizing invasive cancer is nearly 100%, yet specificity values vary [33]. A study investigating preoperative imaging techniques found that MRI had a detection rate comparable to that of US (which is unaffected by tumor size in either case) and a sensitivity rate higher than that of both mammography and US [33]. Also, MRI can better detect intraductal spread than can US or mammography [35]. MRI also shows promise in screening women with familial or genetic predispositions to breast cancer [36]. Results of these two studies can be found in Table 1.

With respect to DCIS, the sensitivity of MRI ranges from 40% to 100%, with some authors reporting less sensitivity than mammography. With respect to invasive lobular carcinoma, sensitivity is 93.3% (through meta-analysis), with a correlation with pathologic abnormalities ranging from 0.81 to 0.97 [37], making it more sensitive than mammography. In correlation with mammography, MRI yielded a sensitivity rate of 88.1% and a specificity of 67.7% prior to biopsy in a study by Bluemke and colleagues [38]. This study also corroborated the results of the aforementioned studies, finding greater sensitivity with respect to invasive cancer than DCIS.

Whereas US-guided biopsies are easy, MRI-guided biopsies are more difficult, requiring special MR-compatible equipment such as needles [33]. Also, with MRI, the lateral side of the breast is the only accessible side in most systems [33]. It is difficult to ensure the removal of the lesion [33] using needle-based techniques compared with conventional mammogram- or ultrasound-guided biopsies. 

MRI is the best technique for postchemotherapy imaging. Accuracy may vary with the degree of response to chemotherapy [32, 39]. A study by Partridge et al. [40] found a correlation with pathologic abnormalities of 0.89 for MRI and 0.60 for clinical measurements, confirming the value of the use of MRI in patients who have undergone neoadjuvant chemotherapy. The MRI technique is also sensitive in imaging silicone breast implants. A study found that the rates of sensitivity and specificity for detection of silicone implant rupture were 76% and 97%, respectively, making it superior to mammography and ultrasonography for that indication [34]. MRI has better resolution and less operator dependence than US [33]. Also, it does not use radiation, making it safer than those modalities that do, thus allowing use with pregnant patients, although the effect of contrast media on the fetus is not known [33]. On the other hand, MRI is costly, without a clear notion of cost effectiveness [33]. Its contrast agent can also affect benign lesions [33].

One must note the paucity of standardized indications for MRI use, procedure protocol, or interpretation [33]. Orel [39] states that MRI “should never be used in place of a full mammographic and sonographic evaluation”. Lalonde et al. [41] advise that “MRI should not be used to decide whether a lesion seen on conventional imaging should undergo a biopsy” and that MRI should not be used to categorize a lesion as BIRADS 3 or 4, meaning probably benign or suspicious, respectively. A recent study indicated that the routine use of MRI in women already identified as having breast cancer increased the detection of synchronous disease [42]. Although commonly used to screen high-risk populations, as we gain more clinical experience with MRI, its role in diagnosis is becoming clearer, and the likelihood that it will be a successful general screening modality is decreasing.

### 2.6. Nuclear Medicine

Nuclear medicine techniques yield functional images based on molecular properties. They do not have unfavorable effects stemming from breast density [15]. Also, these techniques require little or no breast compression. Nuclear medicine techniques are expensive and expose the patient to radiation yet show promise in detecting cancer, especially in high-risk patients.

#### 2.6.1. Radioimmunoscintigraphy

In radioimmunoscintigraphy (RIS), tumor-associated antigens are targeted by an injected radiopharmaceutical agent [43, 44]. Identification is based on differences in antigen expression in normal and cancer cells. Although earlier work used ^131^I and ^111^In, the most commonly used label today is ^99m^Tc. Antigens targeted by this technique include carcinoembryonic antigen, polymorphic breast epithelial mucin antigen, and TAG72 antigen [44]. It can also be used for receptor imaging, using somatostatin receptors [44]. RIS can also be performed using perfusion agents, including thallium-201, ^99m^Tc-sestamibi, ^99m^Tc-tetrofosmin, ^99m^Tc-methylene diphosphonate, and ^99m^Tc-diethylene triamine penta-acetic acid [15, 43]. RIS is associated with high cost and with moderate rates of sensitivity and specificity, indicating that it is not a good screening method [44].

#### 2.6.2. ^99m^-Tc-Sestamibi Scintimammography

Because of its strong attraction to the negatively charged mitochondria, the lipophilic ^99m^Tc-hexakis-2-methoxy isobutyl isonitrile molecule has become widely used in scintimammography [44]. Its uptake “is due to an increase in blood flow, number of mitochondria and cancer cell membrane hyperpolarization in the tumor and as a function of the expression of the multidrug resistance gene” [43]. The technique of ^99m^-Tc-sestamibi scintimammography (SMM) is best performed “before or after 7–10 days after fine needle aspiration, 4–6 weeks after breast biopsy and at least 2-3 months after breast surgery or radiotherapy” [44]. A multicenter study conducted by Sampalis et al. [45] found sensitivity, specificity, positive predictive, and negative predictive values related to SMM of 93%, 87%, 58%, and 98%, respectively. Similarly, a meta-analysis of 64 unique studies published between 1967 and 1999 yielded a sensitivity of 85.2% and a specificity of 86.6% [46]. A comparison of SMM to MRI found that SMM had a higher specificity than MRI, indicating that it could be used clinically to evaluate lesions larger than 1 cm in diameter [47]. A comparison with sonography found that the negative aspects of SMM overshadowed its high detection accuracy, indicating that it probably will not replace sonography [48]. A study by Cwikla and colleagues [49] found that SMM detected more cases of multifocal and multicentric cancer than both mammography and ultrasonography. A study by Hillner [50] estimated that SMM usage in comparison to immediate surgery would decrease costs by 39% and result in 65% of women avoiding biopsy. Khalkhali and Itti [51] recommended SMM for primary cancer detection in patients with dense breasts and in high-risk patients with no abnormalities found by mammography and CBE, detection of cancer recurrence, monitoring response to neoadjuvant chemotherapy, and radionuclide-guided prebiopsy. 

SMM, however, takes longer to perform than US and subjects the patient to ionizing radiation [48]. The technology is associated with a high rate of false-positive responses [46, 48] and low sensitivity for small cancers (<1–1.5 cm) and DCIS [51].

Planar and single-photon emission computed tomography (SPECT) are methods used to image the aforementioned radiopharmaceuticals agents. Planar imaging uses a conventional gamma camera to create a 2D image [43]. SPECT uses multiple planar images taken at different angles to reconstruct a 3D image [43]. In fact, a SPECT system dedicated solely to breast imaging has been developed [52].

#### 2.6.3. Positron Emission Tomography

Positron emission tomography (PET), generally using ^18^F-fluorodeoxyglucose (^18^F-FDG or simply FDG), uses glucose metabolism to detect cancer. This radiotracer has a high lesion-to-background ratio [53]. FDG-PET has a well-established track record for the detection of malignancy, especially metastatic disease in solid organ tumors. PET can also measure methionine metabolism, using L-methyl-^11^C-methionine, and hormone receptors, using 16-^18^fluoroestradiol 17 *β* [44]. Another radiotracer, ^18^F-fluoro-L-thymidine (FLT), has been used to measure thymidine kinase-1 activity, which is associated with S-phase DNA synthesis [54]. FLT uptake is less sensitive to inflammation caused by needle biopsy, providing an advantage in similar situations [54].

Imaging generally occurs 40 to 60 minutes after injection [55], yet Boerner et al. [56] showed that waiting longer increases the detection rate. It has also been found that PET imaging in the prone position with the aid of a breast positioning device improves the modality's cancer detection rate [57]. PET is useful in follow-up examinations, scanning the entire body for recurrence; Lind and colleagues [55] found an average sensitivity of 96% and specificity of 77% in their study. Like SMM, PET has difficulties detecting small primary cancers [58], making it unlikely to become a useful screening modality.

## 3. Experimental Techniques

### 3.1. Advanced Applications of Digital Mammography

In addition to CAD, the ability of FFDM to detect lesions can be improved in a number of ways. We discuss tomosynthesis and contrast-enhanced digital mammography.

#### 3.1.1. Tomosynthesis

In tomosynthesis, multiple images (at least eight) are obtained using FFDM while the X-ray tube changes angles [59]. In some types of tomosynthesis, the detector remains in place (yielding a more restricted view); whereas in others, the detector follows in rotation. Exposures are made as the angle changes. Each exposure is done with a low dose (each about 10% of a normal mammogram), either taken in a step-and-shoot method or in a continuous method involving X-ray pulses [60]. As the X-rays change angles, objects within the breasts change heights, allowing for computer reconstruction. This technique gives rise to three-dimensional (3D) images that can be viewed as individual slices or in dynamic cine [60]. Tomosynthesis requires less breast compression time and pressure than a normal mammogram, providing the patient greater comfort [60]. Tomosynthesis systems do not require moving parts near the breast or abdomen, can be used with minor alterations to existing mammographic systems, and are able to take conventional mammograms as well [59]. Tomosynthesis can detect 16% more lesions than can be seen on a normal mammogram and has been reported to reduce false-positive results by 85% [61]. Unfortunately, individual frames contain more noise than normal FFDM images [62]. Tomosynthesis is starting to be seen in breast screening centers, but more clinical experience is needed to determine if it will become a relevant screening test or if it will become the diagnostic test of choice after a screening mammogram shows an abnormality. In addition, as Good et al. [62] note, tomosynthesis requires a significant amount of experience reading and interpreting the images. Without an FDA approved breast tomosynthesis device, a great deal of published sensitivity and specificity data, or enough clinical trials to determine its best use, this modality remains at what Dobbins III [63] refers to as a “translational crossroads” between the experimental and clinical stages.

#### 3.1.2. Contrast-Enhanced Digital Mammography

Two types of contrast-enhanced digital mammography are available: temporal subtraction and dual energy.

In temporal subtraction contrast-enhanced digital mammography, an initial FFDM mask image is obtained, then a contrast agent (generally iodine-based) is injected intravenously, and then multiple X-ray images are obtained [13, 64]. Then the image undergoes temporal subtraction. The precontrast mask image is subtracted from the postcontrast image [13, 64].

In dual energy contrast-enhanced digital mammography, two X-ray images are taken after a contrast agent (generally iodine-based as well) is injected intravenously. One X-ray image is high energy, which excites the contrast agent, while the other is below this excitation threshold [64]. The lower energy image is then subtracted from the higher energy image, yielding an X-ray image with greater contrast that is easier to interpret.

Contrast-enhanced digital mammography has a high contrast-to-noise ratio, reducing the visibility of microcalcifications [64]. Also, the iodine-based contrast medium requires a high-energy X-ray. New contrast agents (bismuth-based for temporal or zirconium-based for dual energy) could allow for lower energy X-rays, but this result would involve development of a contrast agent used exclusively for mammograms, which is expensive [64]. Also, the subtraction process may lead to the appearance of artifacts [64]. Contrast-enhanced digital mammography can be combined with tomosynthesis. Known as contrast-enhanced digital breast tomosynthesis, this technology has not yet been extensively tested. One study by Chen et al. [65] proposes that these two techniques can be combined, thereby uniting the strengths of each method, to characterize the morphologic and vascular natures of breast lesions. This technology is sensitive to motion, but motion can be decreased by decreasing the length of the procedure [65]. A great deal more work needs to be done in the development of this tool.

### 3.2. Advanced Applications of Ultrasound

#### 3.2.1. Sonoelastography

In sonoelastography (SE), ultrasound images are taken while breast tissue is displaced. Displacement methods include compression, vocal fremitus low-frequency vibrations (the patient hums a pitch), and radiation force [15, 23]. Breast tissues vibrate or compress differently based on their firmness, which permits one to locate masses. Researchers have reported varying results. Some indicate positive results; others conclude that the performance of SE is worse than that of conventional US [23, 66].

#### 3.2.2. Tissue Harmonic Imaging

Tissue harmonic imaging (THI) has two types: narrowband and wideband. In narrowband THI, filters remove the fundamental frequency from echoed harmonics resulting from a narrowband pulse. In wideband THI, two inverted pulses are emitted and added together, removing linear components when they return upon echo [67]. Szopinski et al. [67] found that THI increased gray-scale contrast between fatty tissue and breast lesions in 90.6% of 254 lesions. This improvement in contrast was better in fattier breasts [67]. Other studies indicate that THI may contain data not obtained using B-mode US, suggesting uses other than image enhancement [23].

#### 3.2.3. Ultrasonic Spiral Computed Tomography

A study by Azhari and Sazbon [68] suggests that ultrasonic spiral computed tomography (CT), a cross between CT and US, is a feasible form of volumetric 3D imaging. This technology is suitable almost exclusively for breast imaging; however, it has difficulty in detecting tumors located proximal to the chest wall [68].

### 3.3. Advanced Applications of MRI

#### 3.3.1. Diffusion and Perfusion Imaging

Diffusion and perfusion imaging, two emerging forms of MRI, show promise in characterizing breast tumors. Diffusion imaging offers information concerning tissue microstructure by focusing on water protons [69]. Perfusion imaging follows the flow of blood to tissue, showing the microvasculature [69]. Research shows that the values of the apparent diffusion coefficient and the relative tissue blood volumes permit one to distinguish between malignant and benign lesions [69].

#### 3.3.2. Proton Magnetic Resonance Spectroscopy

Proton magnetic resonance (MR) spectroscopy (^1^H-MRS) measures the resonance of protons to provide a spectrum. With this data it can identify a peak of choline, a biomarker of cancer, at 3.23 ppm as opposed to 3.28 ppm in benign breast lesions [70]. Sardanelli et al. [71] found ^1^H-MRS to have a 90% sensitivity rate and an 89% specificity rate, indicating a higher specificity than dynamic MRI. A similar method, ^31^P MR spectroscopy, has also been studied, displaying an increase in phosphomonoesters in breast cancer tissue but with a poor ability to distinguish between malignant and benign tissues [71]. With lipid and water suppression, this technology could be used to gain information about breast tumors. Indeed, the software is now widely available on the newest MRI machines.

#### 3.3.3. MR Elastography

In MR elastography, electromechanical drivers vibrate the breast, generating acoustic shear waves, which are then imaged by MRI [72]. Through this process, stiffness can be measured. McKnight et al. [72] found that breast carcinoma displayed a mean shear stiffness that was 418% greater than that of surrounding breast tissue. This method could be used in the future for the detection, and perhaps characterization, of breast cancer.

### 3.4. Computed Tomography

A new form of CT has been created at the University of California, Davis Medical Center specifically for breast scanning. The 360° scan, which takes 16.6 seconds, obtains about three hundred 512 × 512 images of each breast, creating 3D images [73] that allow analysis by the slice. This technology requires no breast compression because of its specific design. Also, CT images do not experience the distortion seen in MRI, and regular biopsy needles can be used [73]. An algorithm has been created that classifies breast tissue as skin, fat, and glandular tissue. With this algorithm, Nelson et al. [74] were able to dispel the common perception that the breast is composed of 50% fat and 50% glandular tissue; rather it is approximately 70% fat and 30% glandular tissue. This algorithm showed 97.7% agreement with the findings of a radiologist [74].

Whereas normal CT subjects the chest organs of patients to radiation, this specified CT does not; in fact, its average glandular dose of radiation is 6.4 mGy per breast, which is not much more than that of a two-view mammogram [74]. CT scanning can be enhanced using iodine-based contrast agents (similar to the aforementioned). The contrast of CT images can also be heightened by diffraction-enhanced imaging (DEI), which, after lowering the radiation dose of current trials, could be used clinically [75]. Boone et al. [73] predicted that tomosynthesis, which uses a limited angle to create a thick slice image, will “outperform breast CT for microcalcification detection, while breast CT will likely outperform tomosynthesis for soft tissue (mass) lesion detection.” Boone and colleagues [73] also estimated that breast CT will cost less than half the price of an MRI-based biopsy. CT requires its subject to remain still, while holding her breath for the duration of the scan. A move toward a 9-second scan, as proposed by Boone and colleagues [73], should reduce patient discomfort and the appearance of motion artifacts. Other research has looked into cone-beam volume CT breast imaging, a technology that uses a rotating X-ray to scan one breast at a time while it hangs through a hole in a table [76]. This technology also delivers an amount of radiation comparable to that from a normal mammogram and obtains a 3D image with much greater contrast than a normal mammogram. This approach may improve imaging without increasing radiation exposure and without compression, but it will not reduce the cost or improve the access to screening associated with mammography.

### 3.5. Advanced Applications of Nuclear Medicine

#### 3.5.1. Positron Emission Mammography

A more breast-specific PET technique known as positron emission mammography (PEM) or breast-specific gamma imaging focuses on imaging the breasts rather than the entire body. PEM costs less than conventional PET [43]. It has higher spatial resolution [43, 77], requires a lower dose of FDG [77], and takes less time to perform [77]. PEM has also been reported to be better at detecting DCIS and small cancers than other nuclear medicine techniques [78, 79]. Still, one encounters difficulty imaging the far posterior area of the breast, and the technique suffers from high false-positive rates due to fat necrosis at prior biopsy locations [77]. PEM is best for screening high-risk patients [79]. Data concerning this technique are summarized in Table 2.

#### 3.5.2. PET/CT

The PET and CT systems have recently been combined, allowing for both imaging techniques without repositioning the patient [53]. This modality costs more than each individual system yet requires less time to acquire anatomical and molecular data simultaneously [53, 80]. A recent study, using ^18^F-FDG PET/CT for axillary staging, found that the technology had an 83% accuracy, 58% sensitivity, and 95% specificity; despite its limited sensitivity, its diagnostic accuracy is comparable to that of US [81]. At present, no studies of PET/CT as a screening modality exist.

### 3.6. Diffraction Techniques

#### 3.6.1. Diffraction-Enhanced Imaging

In DEI, a crystal, called the analyzer crystal, is placed between the object and the detector (digital or X-ray film) based on Bragg geometry. A synchrotron delivers a monoenergetic beam through the object and delivers two images on either side of the rocking curve by changing the angle of the analyzer [82]. The images contain the same apparent absorption data and opposite refraction data. By applying an algorithm, adding pixel by pixel, an apparent absorption image (similar to that of a normal X-ray) can be obtained; by subtracting pixel by pixel, a refraction image can be obtained [83]. A peak image can also be obtained, which is recorded at the peak of the rocking curve [83]. Breast tissue microstructures are seen with the best clarity and contrast in refraction images, followed by peak images, and then apparent absorption images [83].

DEI requires a short exposure time and has higher spatial resolution than B-mode ultrasonography, MRI, and CT [83]. Though DEI images are clearer using X-ray film and viewed by an optical microscope, it is more suitable to use digital media [83]. One study of seven cases found that six (86%) showed “enhanced visibility of surface speculation that corresponded with histopathologic information” [84]. Another study, performed by Kiss et al. [85], showed that DEI improved contrast of calcifications by a factor of 19. Studies suggest that with improvements, such as enabling compatibility with usual X-ray sources, DEI should move toward clinical use, detecting early stage breast cancer such as DCIS [83, 85].

#### 3.6.2. Small-Angle X-Ray Scattering

Small-angle X-ray scattering (SAXS) uses the coherent scattering obtained at small angles (between 3° and 10°) to classify tissues [86]. This technique can identify structures as small as 0.1 nm [87, 88]. SAXS can identify collagen fiber patterns, which are related to the spread of cancer. Changizi and colleagues [86] reported the ability to differentiate between normal, benign, and malignant breast tissues. Also, Round et al. [89] found 100% sensitivity associated with SAXS, although from a small sample size. SAXS, once it becomes compatible with conventional X-ray sources, could be used as a clinical diagnostic tool to microscopically image areas of concern in vivo, obviating the need for tissue biopsy [89]. One study suggests that a combination of SAXS and DEI could be used as a diagnostic tool [87].

### 3.7. Raman Spectroscopy

In the inelastic scattering process known as Raman spectroscopy, a laser is used to excite photons, causing energy transfers between vibrational modes [90]. The optimum wavelength for this type of spectroscopy is near infrared, from 785 nm to 840 nm [91]. The Raman spectra peaks correspond to different molecules. Whereas normal mammary spectra primarily contain peaks associated with lipids, tumor-containing mammary glands show an increase in peaks, indicating proteins, and a decrease in those indicating lipids [90, 92].

Raman spectroscopy is accurate, displaying an ability to identify 91% of tumor spectra correctly [92]. It is noninvasive and can be performed in real time inexpensively while consuming very little time [92, 93]. Also, results are not significantly affected by breast density or menopause [90]. Raman spectroscopy can also detect preneoplastic changes by detecting chemical changes in the tumor bed [92]. One common form of Raman spectroscopy is spatially offset Raman spectroscopy (SORS), which uses backscattering to identify individual components in the sample [93]. Recent work by Stone and Matousek [91, 93] has focused on transmission Raman spectroscopy rather than SORS. This technique cannot be used to identify the depth of the tumor yet it can be used to identify calcified material and classify its composition at greater depths than SORS [93], a maximum depth so far of 2.7 cm using a dielectric filter in a phantom breast made of porcine tissues [91]. Transmission Raman spectroscopy can be used together with SORS, ultrasonography, or mammography to locate calcifications. Raman spectroscopy shows potential for rapid breast cancer detection in tissue samples and, perhaps eventually with improvements, as an adjunct to other screening modalities.

### 3.8. Diffuse Optical Imaging

Diffuse optical imaging (DOI), or optical mammography (OM), uses near infrared light to detect functional abnormalities in tissue [94]. Detected properties, many of which are linked to angiogenesis and hypoxia, include hemoglobin concentration, blood oxygen saturation, water content, and lipid content [94, 95]. About 85% of breast lesions can be discovered using DOI [94]. This technique is “non-invasive, non-ionizing, low-cost … [and] requires little or no breast compression” [94].

DOI comprises two types: transillumination and tomographic. In transillumination DOI, the detectors lie opposite the sources with the breast residing in the middle, resulting in 2D images [94]. In diffuse optical tomography (DOT), a 3D map is obtained through sources and detectors placed on the surface of the breast at different angles [94, 95].

DOI methods can be improved by the use of contrast-enhancing agents. The most commonly used agent is indocyanine green, which is a safe near infrared-absorbing fluorescent dye [94, 95]. DOI can also be improved by adding sound through the processes of acousto-optical tomography (AOT), also known as ultrasound-modulated optical tomography, and photoacoustic tomography (PAT), also known as optoacoustic or thermoacoustic tomography. These methods add the resolution advantage of ultrasound to the contrast advantage of optical imaging [96]. An ultrasound wave is sent into the tissue in AOT; whereas in PAT ultrasonic transducers measure photoacoustic waves that have been excited by a laser [96]. These techniques can aid molecular and functional imaging.

DOI shows promise for detecting cancers early, based on molecular changes. DOI is performed through three different types of systems: time domain, frequency domain, and continuous wave. From the data acquired by these methods, algorithms create images, whether 2D or 3D.

#### 3.8.1. Time Domain

Time domain (TD) systems use picosecond pulses of light shone upon the breast, which are detected as they exit [94]. The temporal distribution (time of flight) is measured, and properties of the tissue can be determined from this distribution [94, 97]. This process can be bettered through time-gating [97]. The equipment used in this technique is expensive [94, 97].

#### 3.8.2. Frequency Domain

Frequency domain (FD) systems continuously shine light on the breast while the amplitude of its frequency is modulated by tens to hundreds of megahertz [94]. Amplitude decay and phase-shift measurements provide information concerning the tissue's properties [94, 97].

#### 3.8.3. Continuous Wave

Continuous wave (CW) systems determine tissue properties measuring the attenuation of light across the breast [94]. This light is delivered continuously at a constant or low frequency modulated amplitude [94]. Although this method is cheaper and simpler than TD and FD, it cannot determine the internal absorption and scattering properties, and it is very sensitive to variation in surface coupling [94, 97].

### 3.9. Electrical Impedance Scanning

Electrical impedance scanning (EIS), also known as electrical impedance tomography (EIT), measures multiple electrical properties of breast tissue and creates an image. It is meant for discovering nonpalpable lesions [98]. The cell membrane is primarily capacitive but displays conductivity in its semipermeable function. EIS measures conductance at low frequencies (<1000 Hz) and capacitance at higher frequencies [99]. These properties factor into the impedance value. Most EIS systems operate at high frequencies because, at low frequencies, electrode impedances affect the constant nature of the input current [100]. Cancerous and normal tissues have different electrical properties, yet the values overlap at a point [99].

Many electrical impedance scans have been performed using the Siemens TransScan TS2000 and TS2000ED (Early Detection). A similar system is used at Dartmouth College. The systems work by sending a current into the breast and measuring voltages at electrodes placed on the surface. The measurements are then analyzed by a computer algorithm. EIS is noninvasive, relatively inexpensive, and risk free [98, 100, 101]. Scans usually take about 15 minutes, causing the patient little discomfort. It can be performed in vivo or in vitro, though in vitro measurements must be made soon after the death of the tissue [99]. Unlike mammograms, EIS works well with dense breasts. It has also been shown to detect extremely small lesions that other methods might miss [102]. These scans are subject to variation caused by the patients' hormonal changes as well as other factors including “superficial skin lesions, poor contact, and air bubbles” [103].

Though hopes were high for EIS, studies show contradictory results. Although Kneeshaw et al. [104] found that “EIS is able to differentiate malignant from benign disease associated with clinically occult microcalcification,” Wersebe et al. [105] called the diagnostic accuracy of the scan “mediocre.” Melloul et al. [102] decided that EIS, with its 72.2% sensitivity and 67% specificity, did not improve the detection rate of breast cancer when combined with ^99m^Tc-SSM. In another study, EIS yielded a false-positive rate comparable with that of mammography [106]. Similarly, Szabó et al. [107] could not justify the use of EIS as an adjunct to mammography and ultrasonography; the 86% sensitivity of the TransScan TS2000 was similar to the 87% and 75% sensitivities of mammography and ultrasonography, respectively.

The technology yields images with spatial resolution poorer than that of CT and MRI and “has an intrinsically poor signal-to-noise ratio” [100]. Another detriment is the parasitic capacitances related to the input leads [100]. It was recently found that EIS identifies general breast abnormalities, which can show which women are at risk of developing cancer in the future [108]. Stojadinovic et al. [98] noted that the TS2000ED system “seems better suited to identify women at a high risk of breast cancer, in the absence of a specific lesion that can be localized.” In a later study, in which they found 38% sensitivity and 95% specificity, they furthered this statement by recommending that this technology should be used for screening rather than detection or imaging [101].

Halter et al. [109] recently designed an EIS system that uses very high frequencies (as high as 10 MHz) that can measure impedances with an accuracy of 99.7%. At these frequencies, other properties may be measured. Still, further study is needed to determine the benefit of EIS.

### 3.10. Thermography

In thermography, an infrared scan creates an image by mapping temperature differences across the breasts. Because cancerous tumors obtain nutrients through neoangiogenesis and through already existing blood vessels, the local temperature of the cancerous region is generally higher than that of surrounding tissue [110, 111]. Because “each breast has a particular thermographic pattern than does not change over time, much like a fingerprint,” one can take a baseline and mark any significant changes seen on later images for future analysis [110]. Generally, the procedure consists of taking a series of infrared photographs of the breasts while one breast is cooled. The procedure is then repeated, cooling the other breast. After imaging, the data are analyzed by computer algorithms that compare infrared patterns. The procedure, including the imaging process, takes about 15 minutes [112]. Thermographic systems include the BCS2100 and the BreastScan IR.

Infrared thermography has many potential benefits. It is a noninvasive process that consumes minimal time. In one study, Parisky et al. [112] used the BCS2100, finding 97% sensitivity, 14% specificity, 95% negative predictive value, and 24% positive predictive value in a group of 875 subjects, and 99% sensitivity, 18% specificity, 99% negative predictive value, and 27% positive predictive value in a subset excluding lesions deemed to be microcalcifications. In another study, Arena et al. [113] found 98% sensitivity in a study of 67 patients with cancer proven by biopsies.

Thermography works better in certain situations. Imaging is best performed when the female body temperature is most stable: the fifth, twelfth, and the twenty-first days of menstruation [110]. Also, specificity increases when the breast tissue is dense rather than fatty. Larger tumors are more often detected by infrared imaging [114]. This method of detection has its disadvantages. Images are adversely affected by procedural errors, including the amount of cooling and the breast positioning [112]. Larger breasts and dependent areas of the breasts are poorly imaged. Also, if the patient has an asymmetrical body temperature, analysis could result in false-negative and false-positive results. Other limitations include the large size of the computer files and the variations due to age, tumor position, and the aforementioned hormones. Still, this method shows promise for detecting cancer in patients, such as “younger women, men, patients with dense breasts, [and] patients with surgically altered breasts,” that are troublesome for other detection modalities [115].

### 3.11. Compression and Palpation Method

**Figure 1:**
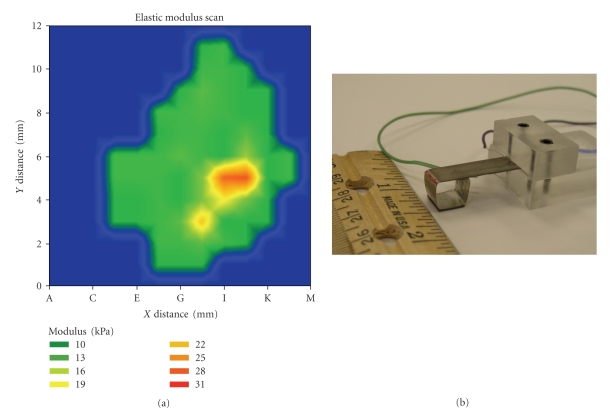
(a) Measured moduli; (b) the piezoelectric finger system. PZT: piezoelectric lead zirconate titanate.

A good clinical breast exam has a high specificity, that is, about 97%. The sensitivity is, however, low because one is unable to palpate small tumors, deep tumors, and microscopic DCIS. The basis of tumor detection and identification in CBE is the fact that malignancies have different elastic and sheer moduli related to the surrounding tissue. Researchers have looked into compression and palpation methods to detect breast cancer. Yegingil et al. [116] created a piezoelectric finger (PEF), a sort of cantilever system, consisting of a stainless steel prod between a top layer of piezoelectric lead zirconate titanate (PZT) for driving and a bottom layer of PZT for sensing. An electric field is applied to the driving PZT layer, causing the finger to bend. The bend induces a voltage across the sensing PZT layer, which is then measured, indicating the displacement. The PEF can measure compression, indentation, shear, and indentation shear, giving the elastic and shear moduli and therefore Poisson's ratio as well [117]. This system can show the size and location of tumors through moduli maps [117]. This system can also determine the depth of the tumor [118]. This system and its measured moduli are modeled in Figure 1. Ex vivo testing on human breast tissue samples resulted in 100% sensitivity and 59% specificity for the detection of breast cancers and DCIS. In vivo human trials are expected within a year.

The PEF has the potential benefits of low cost, portability, results that do not have to be read by a radiologist, and a system that is not user dependent.

A similar system produced by Medical Tactile Inc (Los Angeles, CA), called SureTouch, also mechanically images the breast. This system consists of a probe with a 2D pressure sensor array and an electronic unit that connect to a laptop computer via a USB port [119]. With the software, one can visualize the 2D pressure pattern, the total applied force, and the breast nodule cross-sectional views in real time [119]. The technology has two modes: one for detecting areas of concern and another for characterization [119]. The system works better with a lubricating gel (similar to manual palpation methods) and can detect inclusions farther from the breast surface than a human finger can [119]. It loses reliability when palpating deep lesions in large breasts or mobile lesions in soft breasts, but, considering its “low cost, ease-of-cost, portability, and minimal training required,” the SureTouch system shows potential for clinical or even personal home use [119].

### 3.12. Hair Diffraction

Recent studies indicate that X-ray diffraction of hair could detect breast cancer at a stage earlier than that detectable by mammography [120]. Changes in hair structure, specifically the *α*-keratin fibers that make up the intermediate fibers of hair, display patterns that correspond to those of breast cancer as well as other diseases [120].

Research by James [120] shows 100% sensitivity and 92% specificity. Her findings were confirmed, although with lower sensitivity values, by Corino and French [121].

Requiring only ten hairs, this technique is noninvasive and works with women of all ages [120, 121]. Hairs must be undamaged—slight defects in the hair strands could affect results [120, 121]. Though it cannot locate or image tumors, this technique shows promise as a noninvasive screening tool. A great deal of work remains to be done before it can be used clinically.

### 3.13. Breath Detection

Patients with breast cancer also undergo increased oxidative stress and induction of polymorphic cytochrome P-450 mixed oxidase enzymes, affecting the amount of volatile organic compounds (VOCs) found in the breath [122]. Phillips and colleagues [122] performed breath tests using gas chromatography and mass spectroscopy, finding them to have 94.1% sensitivity and 73.8% specificity for determining the presence of breast cancer compared with results from healthy subjects. In women with abnormal findings on their mammograms but with no cancer detected through biopsy, Phillips et al. [122] found the test to have a sensitivity of 62.7% and a specificity of 84%. This study also reported that breath detection has a negative predictive value of 99.93%, which is higher than that of mammography (99.89%), yet a positive predictive value lower than that of mammography: 4.63% compared to 1.29% [122]. Their data were not affected by patient smoking status [122]. Results indicate that breath detection could help screen for patients with breast cancer without imaging.

#### 3.13.1. Canine Scent Detection

In a recent study by McCulloch and colleagues [123], dogs were trained to detect breast and lung cancer through breath scent, noting that gas chromatography and mass spectroscopy may not be able to find all cancer-related chemicals. The study found 88% sensitivity and 98% specificity in detecting breast cancer and higher values in detecting lung cancer [123]. Though more research is needed, this study shows promise in using canines to screen for cancers. Another study proposed that dogs may also be able to detect the presence of cancer through urine scent [124]; however, the results of this trial were negative.

## 4. Conclusion

Breast cancer is a global problem. It comprises 27% of new cases of cancer in women [2]. With the opportunity for early detection, more lives can be saved. We have summarized the current state of the art in breast cancer screening and early detection. In addition, we have highlighted some emerging technologies that may augment or replace the current modalities.

## Figures and Tables

**Table 1:** Comparative studies of detection techniques. CBE: clinical breast examination; MRI: magnetic resonance imaging; US: ultrasonography.

Study	Value	CBE	Mammography (%)	US (%)	MRI (%)
Preoperative evaluation [35]	Detection rate	—	84.6	97.3	93.7
	Sensitivity	—	22.2	20.6	66.7
	Specificity	—	85.7	85.2	64.2
	Accuracy of intraductal spread	—	50.0	50.0	65.6

Screening in women with familial or genetic predisposition [36]	Sensitivity	17.9	33.3	—	79.5
	Specificity	98.1	95.0	—	89.8

**Table 2:** Results of PEM studies. NPV: negative predictive value; PEM: positron emission mammography; PPV: positive predictive value.

Study	Sensitivity (%)	Specificity (%)	PPV (%)	NPV (%)	Accuracy (%)	Number of lesions
Berg et al. [78]	91	93	95	88	92	92
Rosen et al. [77]*	86	33	90	25	—	20
Levine et al. [58]	86	91	—	—	89	18

*Low values due to small number of true negative cases.
